# Supplementary material for: Advancing Adverse Drug Reaction Prediction with Deep Chemical Language Model for Drug Safety Evaluation
Source: Int J Mol Sci. 2024 Apr 20;25(8):4516. doi: 10.3390/ijms25084516 (PMC11050562; doi:10.3390/ijms25084516)
Supplement: Supplementary file 1 [file ijms-25-04516-s001.zip › Supplementary File S1.pdf]

QUINIDINE GLUCONATE

C=CC1CN2CCC1CC2C(O)c1ccnc2ccc(OC)cc12

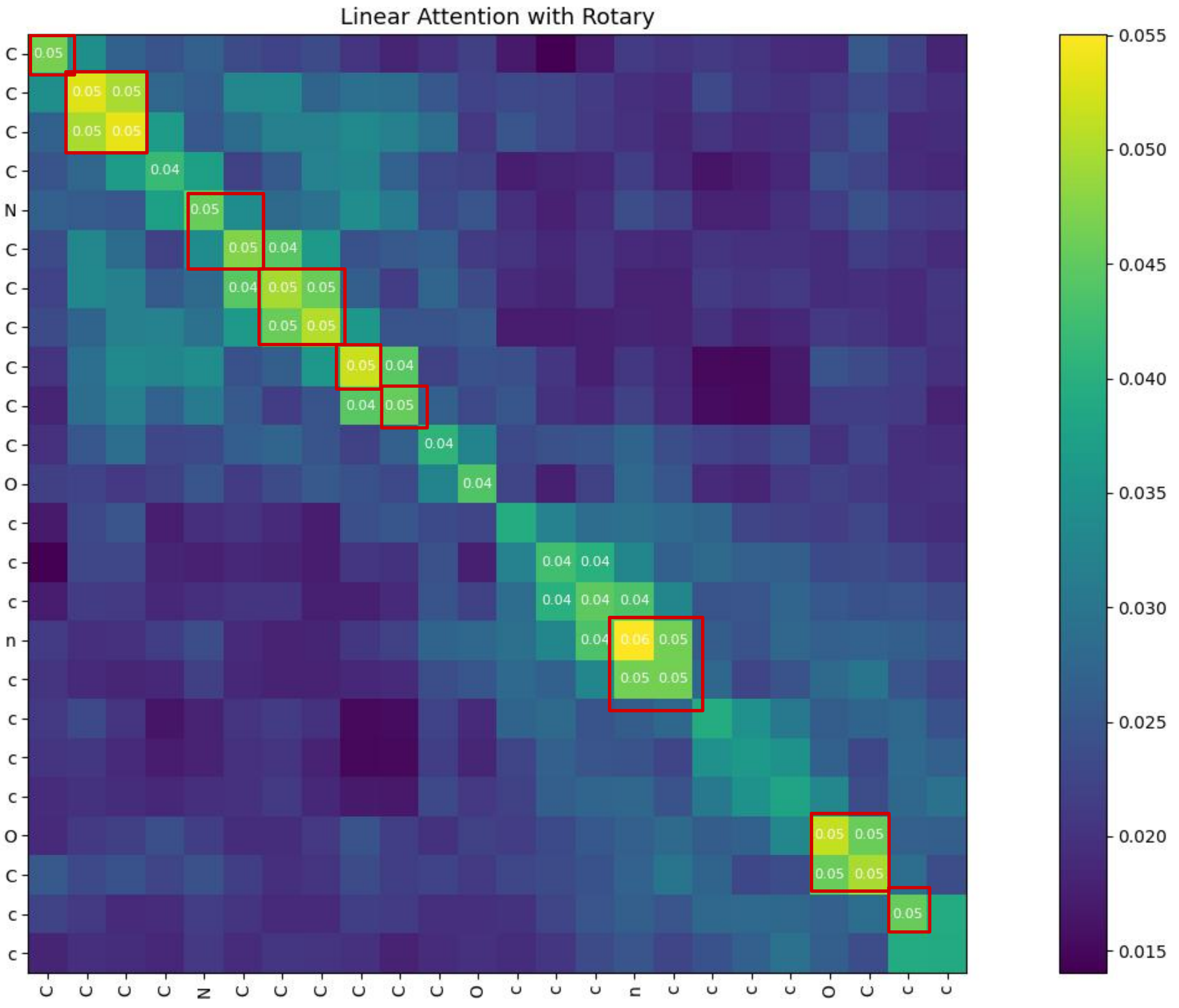

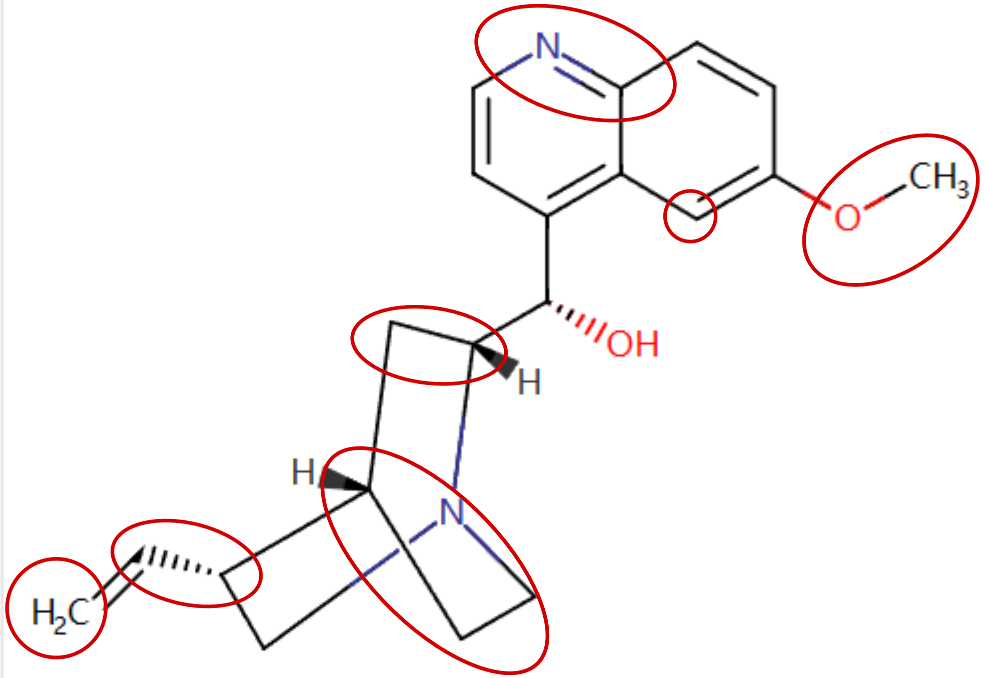

0.06-0.05

VANDETANIB

COc1cc2c(Nc3ccc(Br)cc3F)ncnc2cc1OCC1CCN(C)CC1

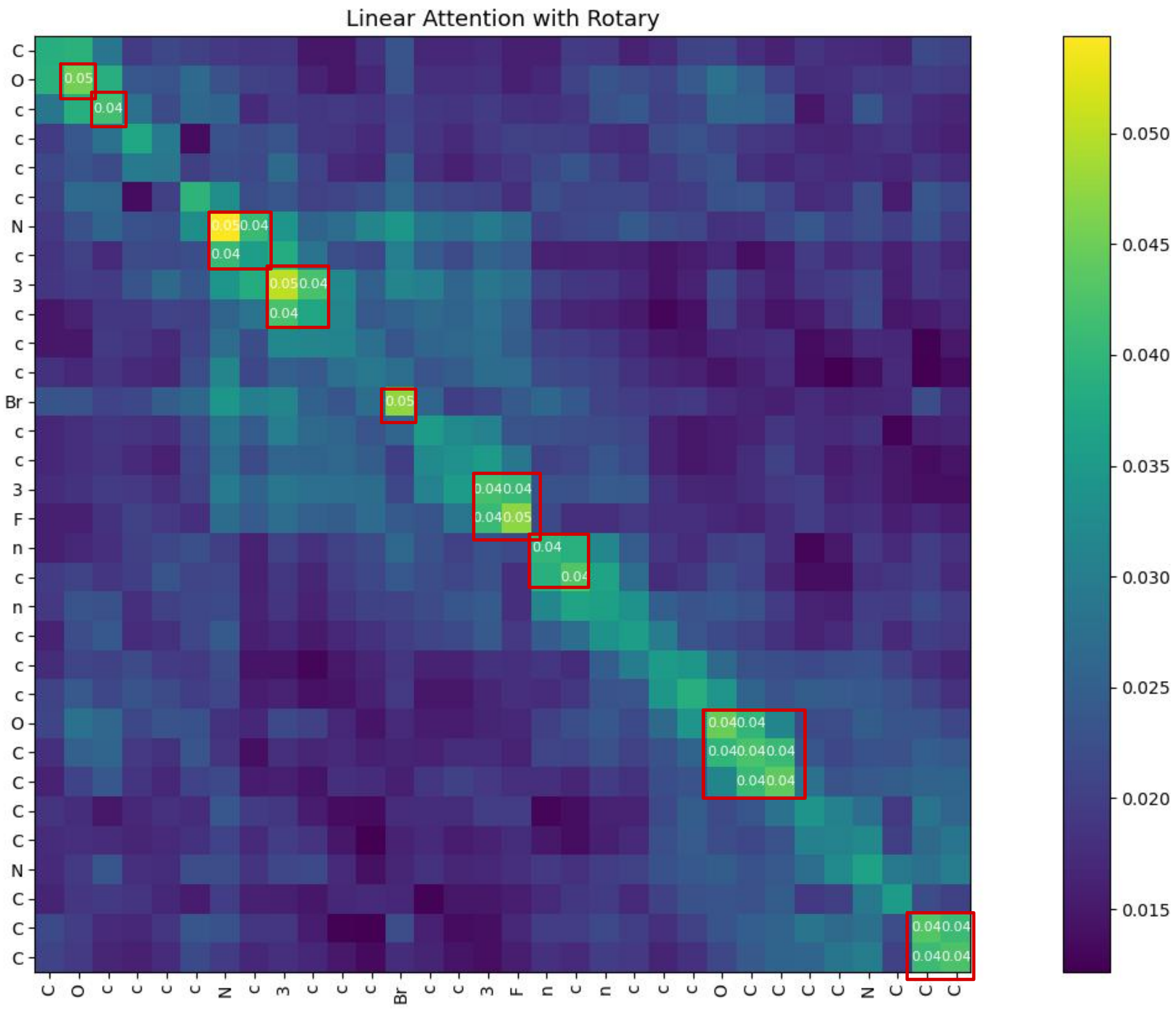

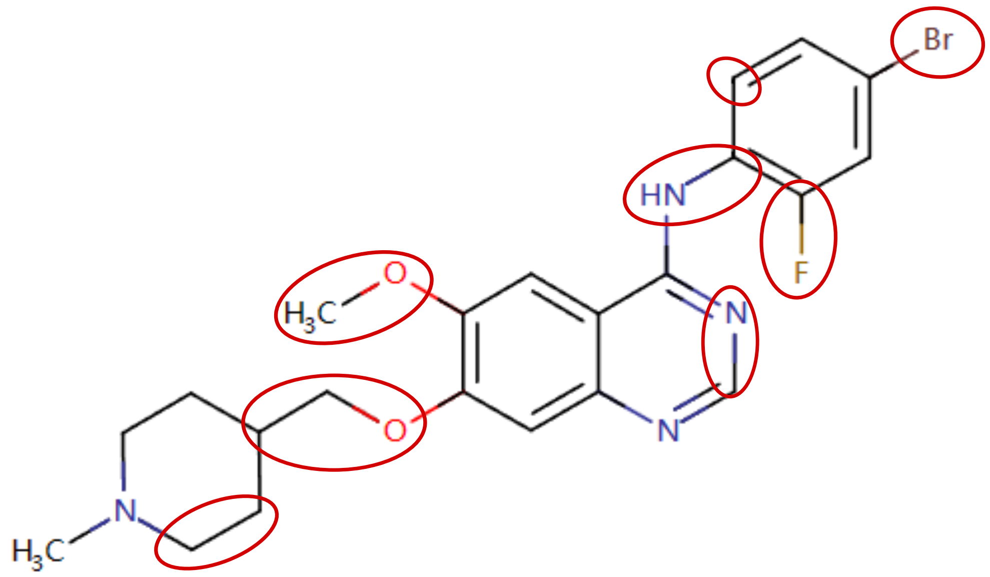

0.05-0.04

IBUTILIDE FUMARATE

CCCCCCCN(CC)CCCC(O)c1ccc(NS(C)(=O)=O)cc1

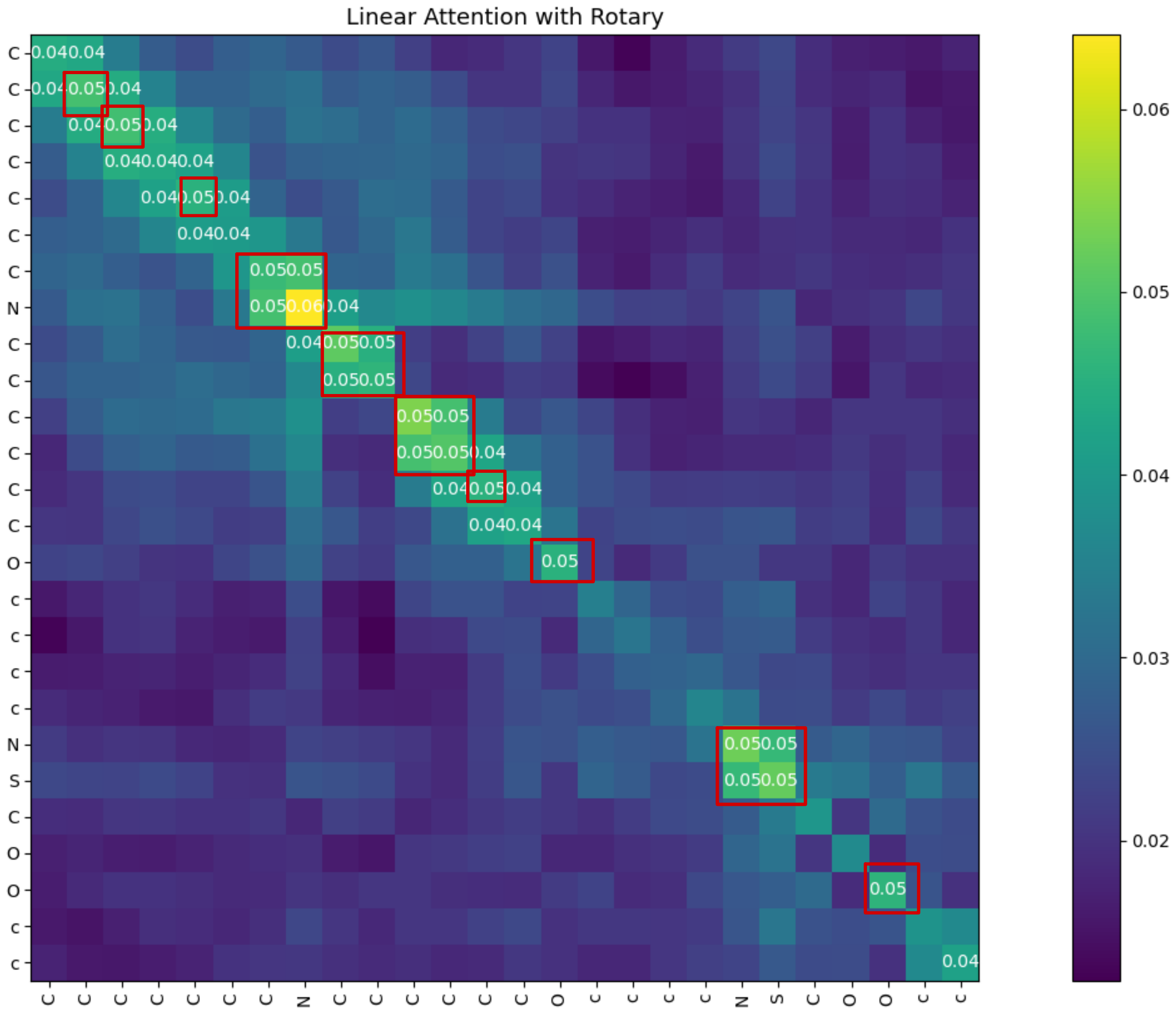

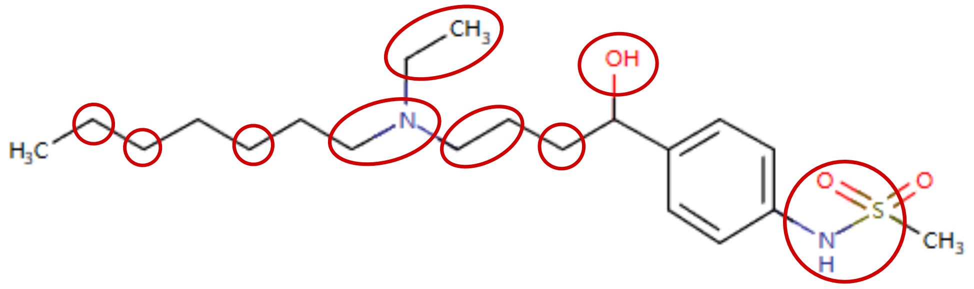

0.06-0.05

DOFETILIDE CN(CCOc1ccc(NS(C)(=O)=O)cc1)CCc1ccc(NS(C)(=O)=O)cc1

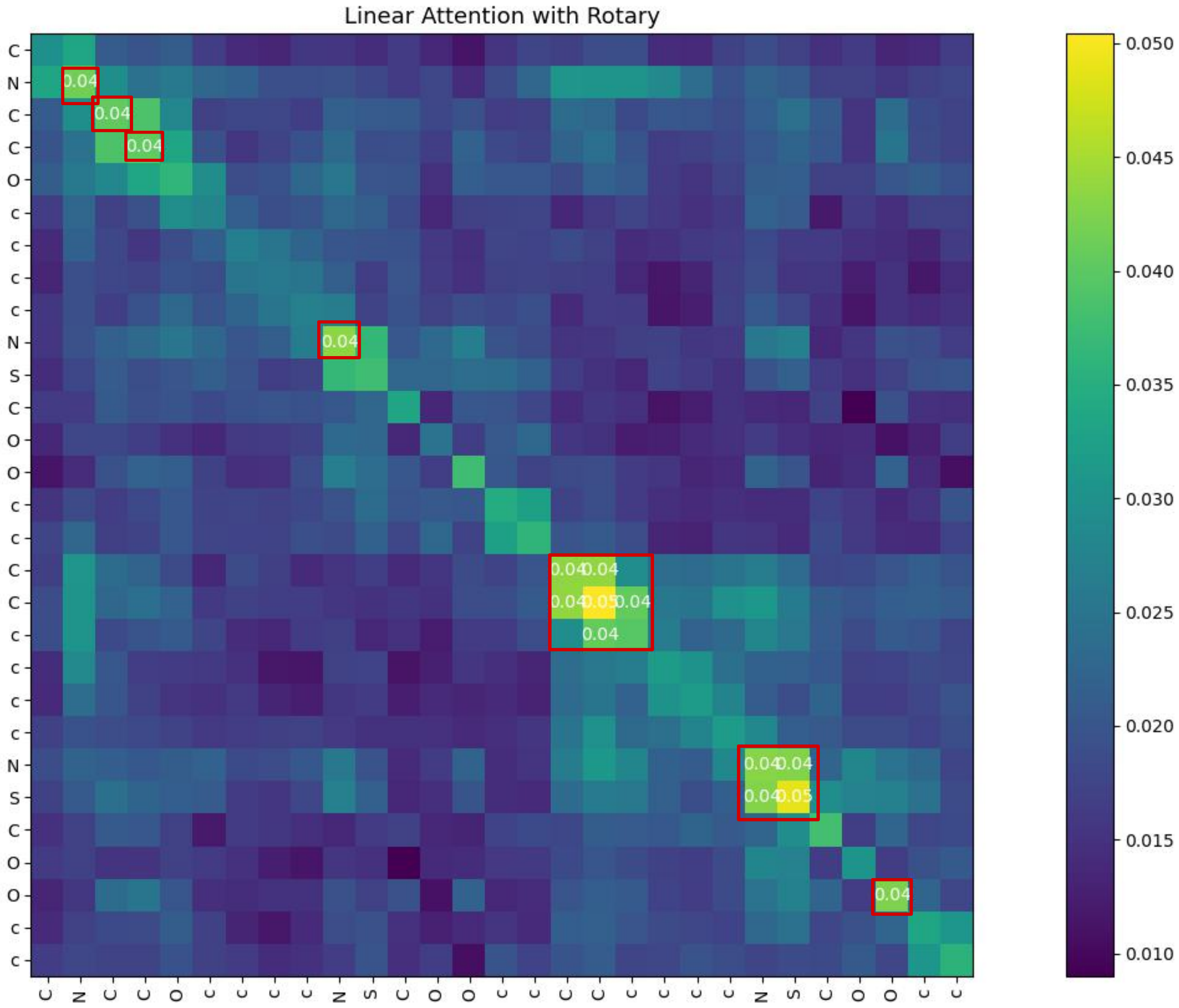

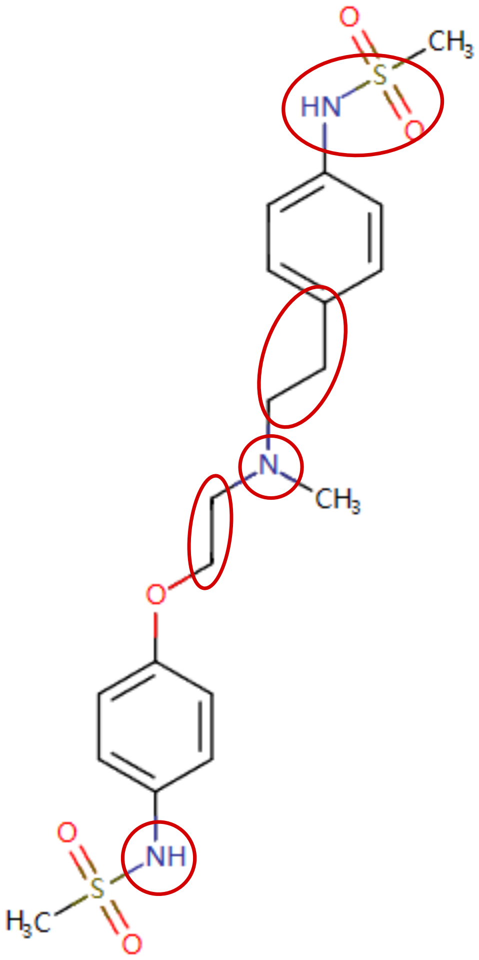

0.05-0.04

DISOPYRAMIDE PHOSPHATE CC(C)N(CCC(C(N)=O)(c1ccccc1)c1ccccn1)C(C)C

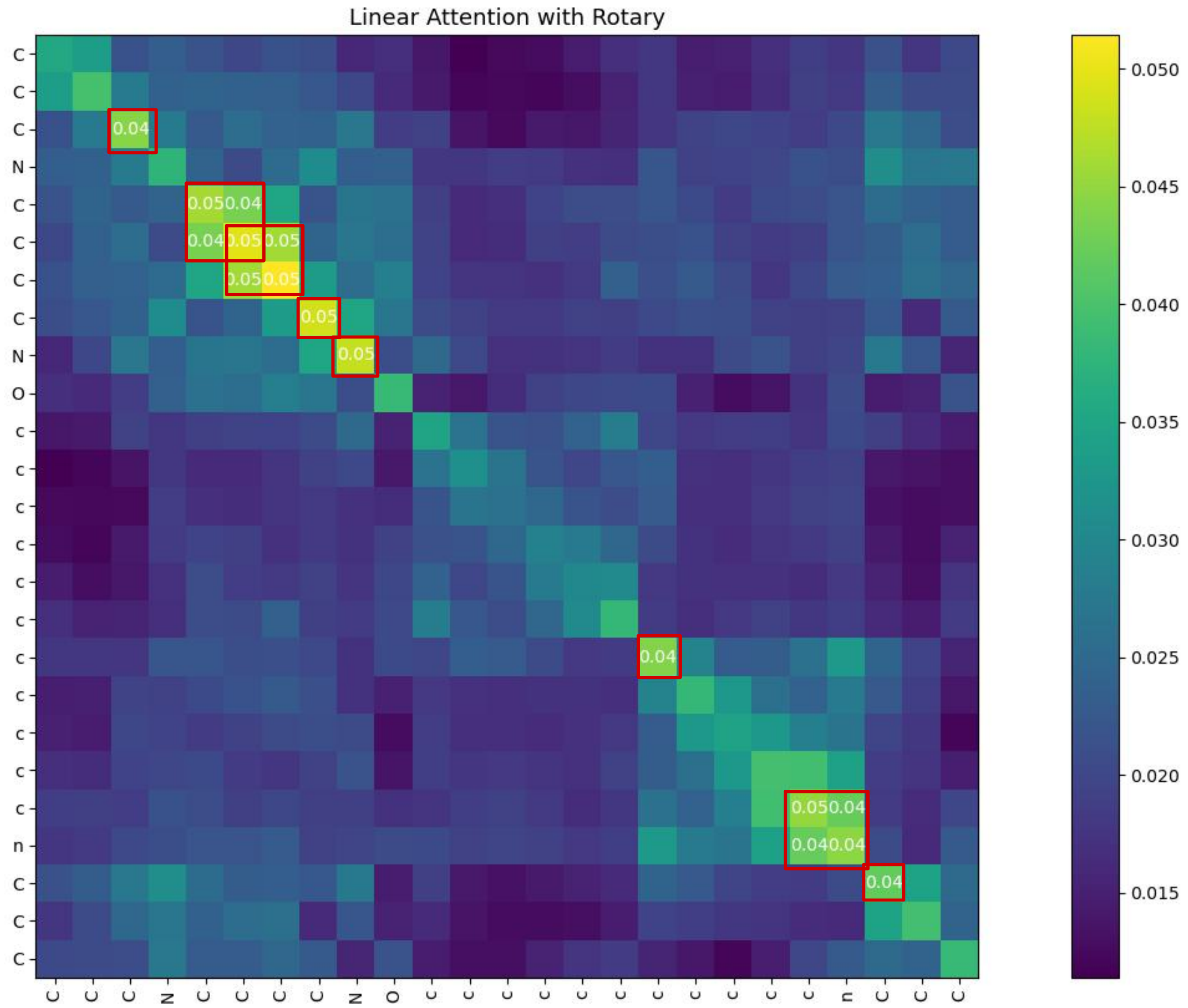

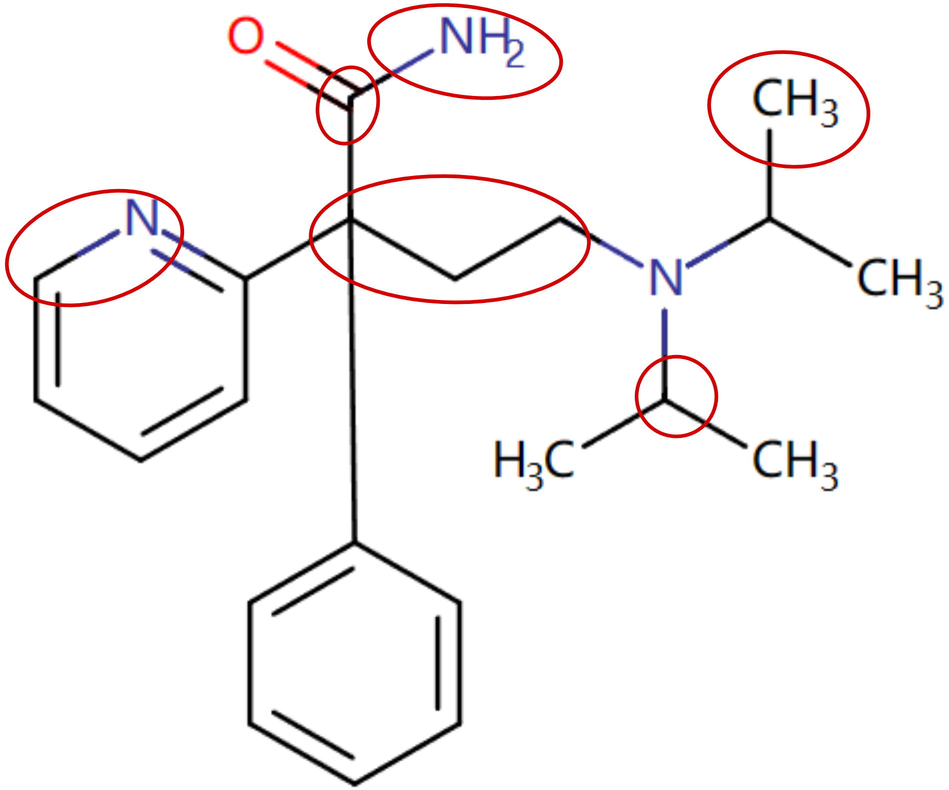

0.05-0.04
