# Supplementary material for: Advancing Adverse Drug Reaction Prediction with Deep Chemical Language Model for Drug Safety Evaluation
Source: Int J Mol Sci. 2024 Apr 20;25(8):4516. doi: 10.3390/ijms25084516 (PMC11050562; doi:10.3390/ijms25084516)
Supplement: Supplementary file 1 [file ijms-25-04516-s001.zip › Supplementary File S3.pdf]

fluvastatin sodium

CC(C)n1c(C=CC(O)CC(O)CC(=O)O)c(-c2ccc(F)cc2)c2ccccc21

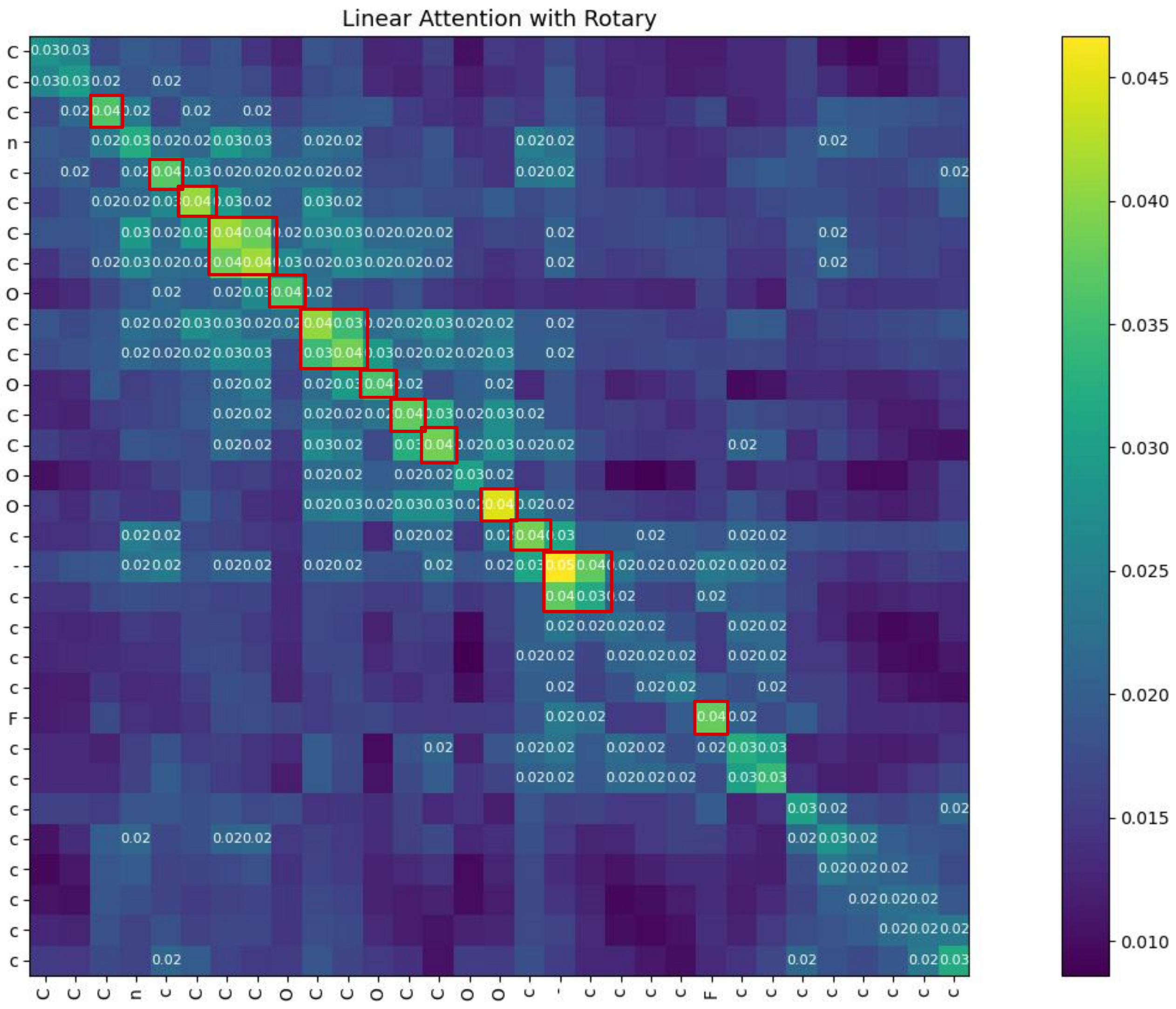

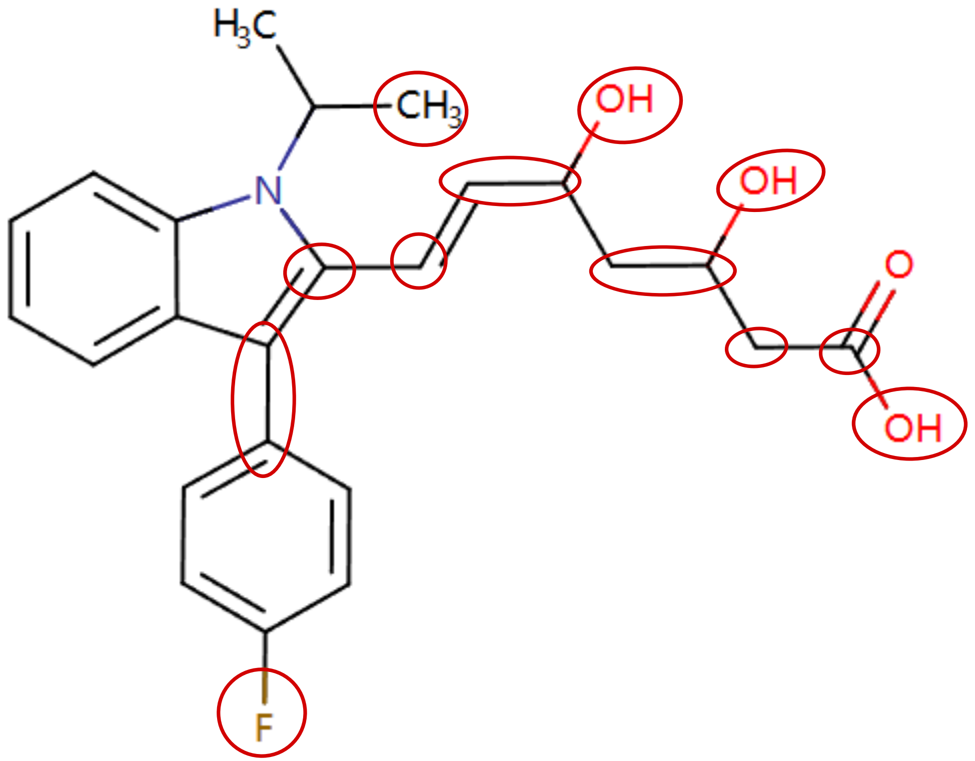

0.05-0.04

lovastatin CCC(C)C(=O)OC1CC(C)C=C2C=CC(C)C(CCC3CC(O)CC(=O)O3)C21

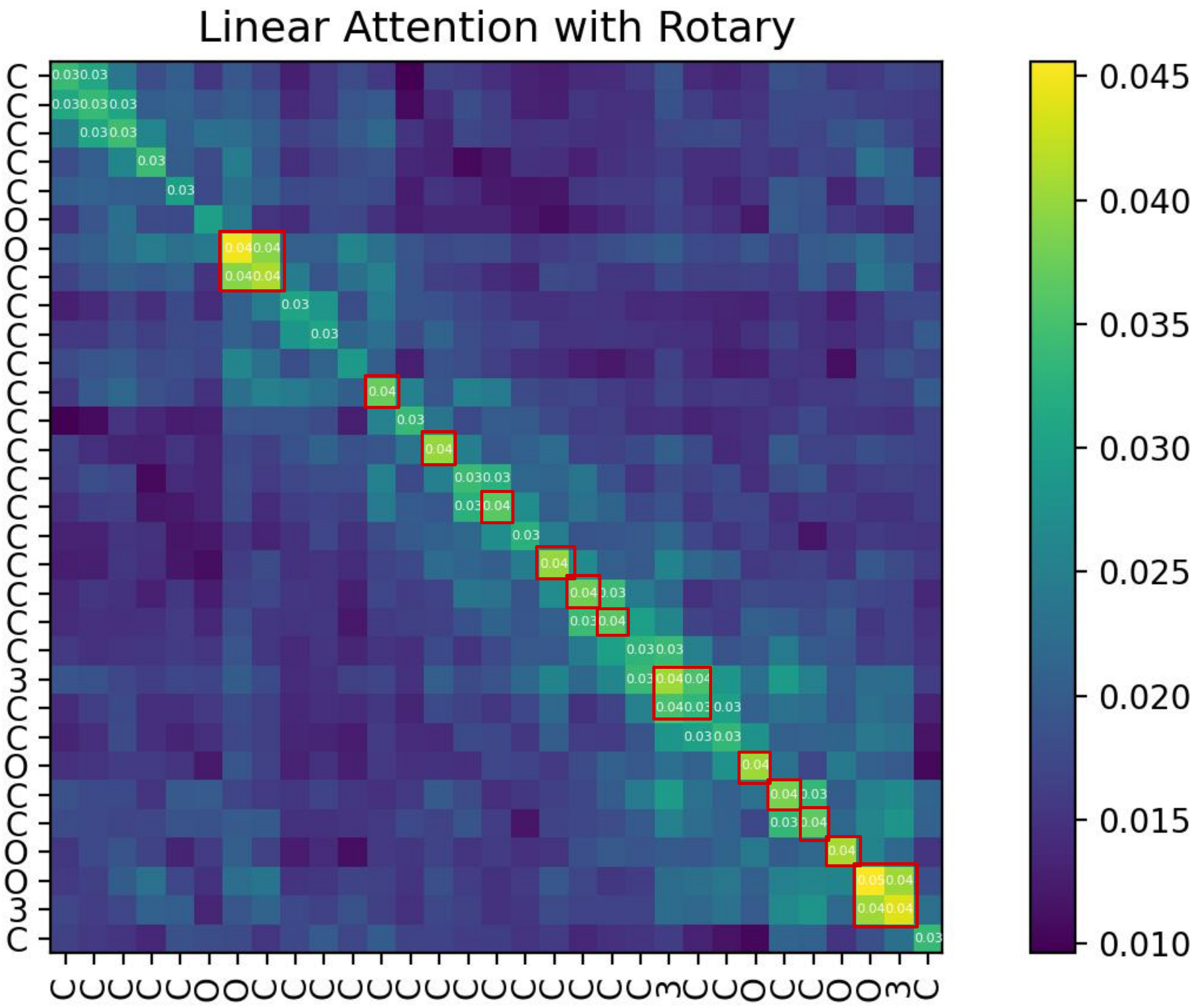

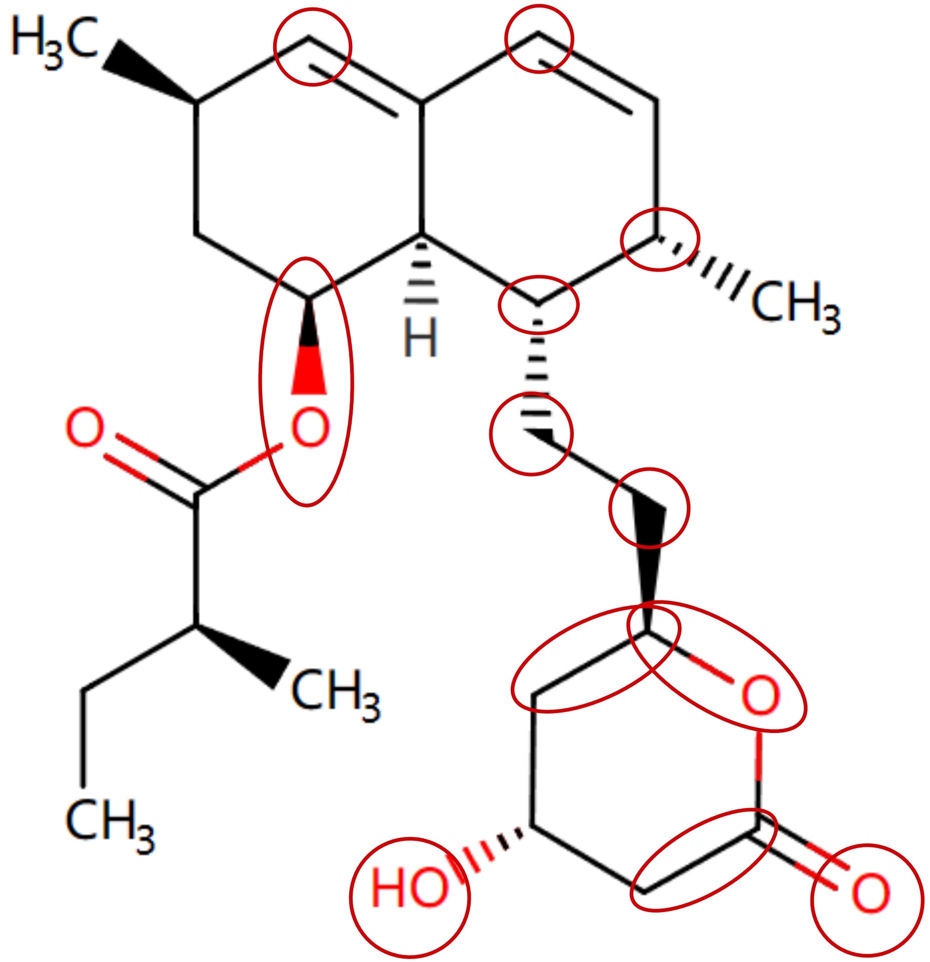

0.05-0.04

pravastatin sodium

CCC(C)C(=O)OC1CC(O)C=C2C=CC(C)C(CCC(O)CC(O)CC(=O)O)C21

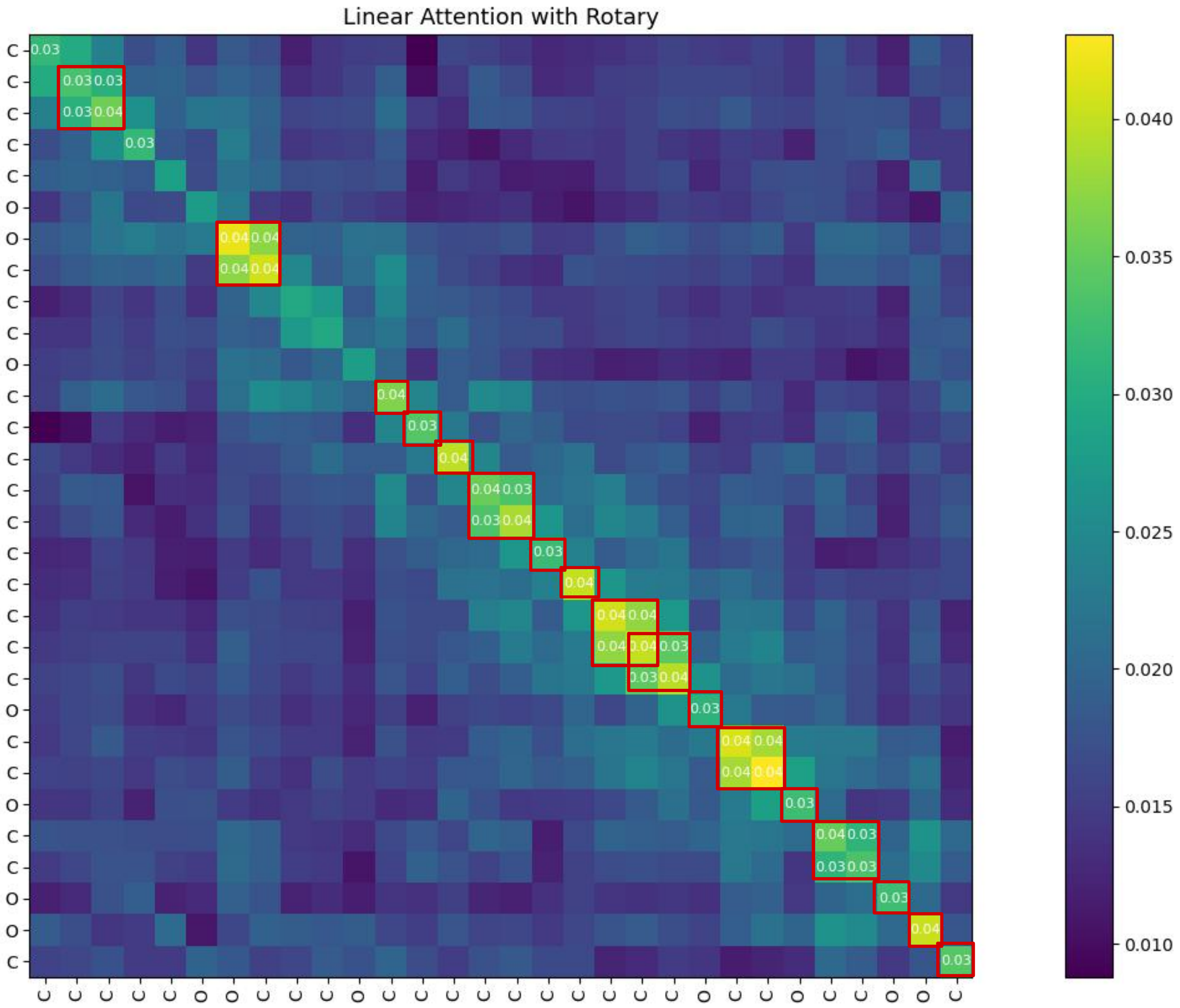

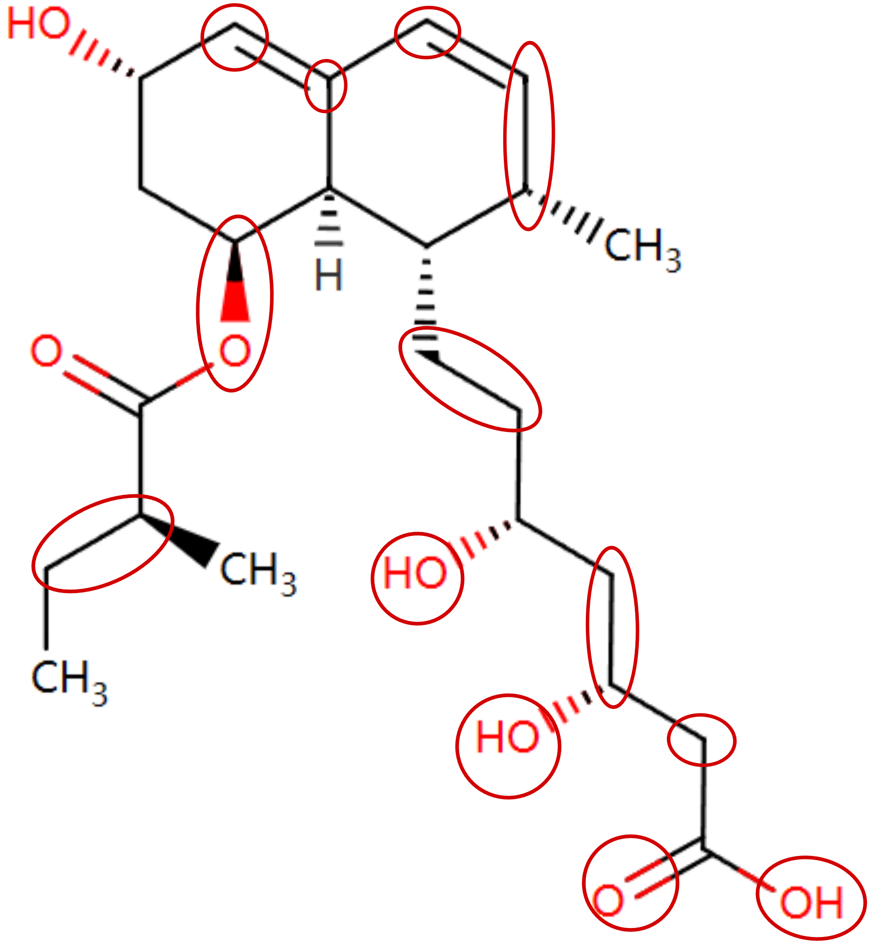

0.04-0.03

atorvastatin calcium CC(C)c1c(C(=O)Nc2ccccc2)c(-c2ccccc2)c(-c2ccc(F)cc2)n1CCC(O)CC(O)CC(=O)O

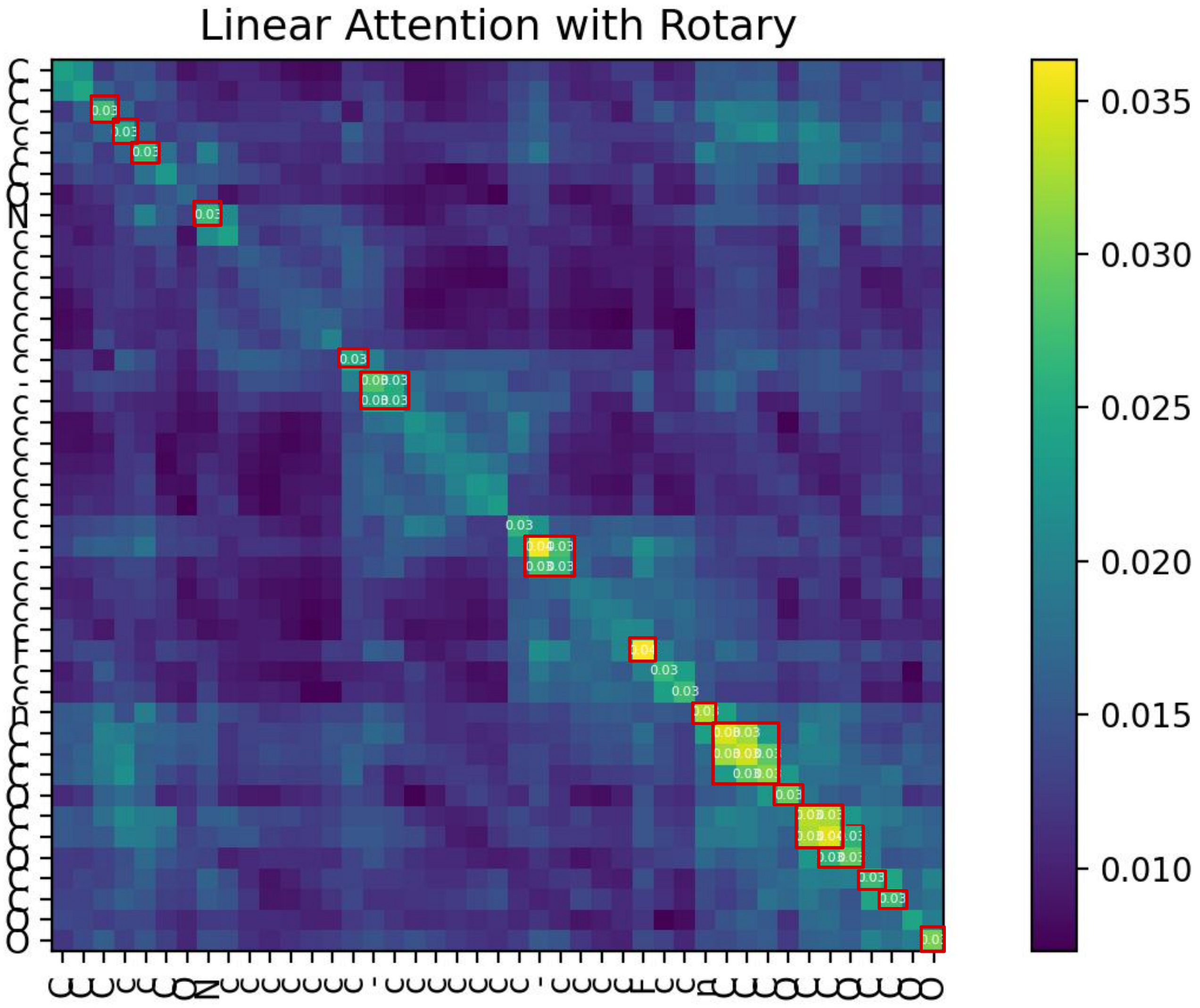

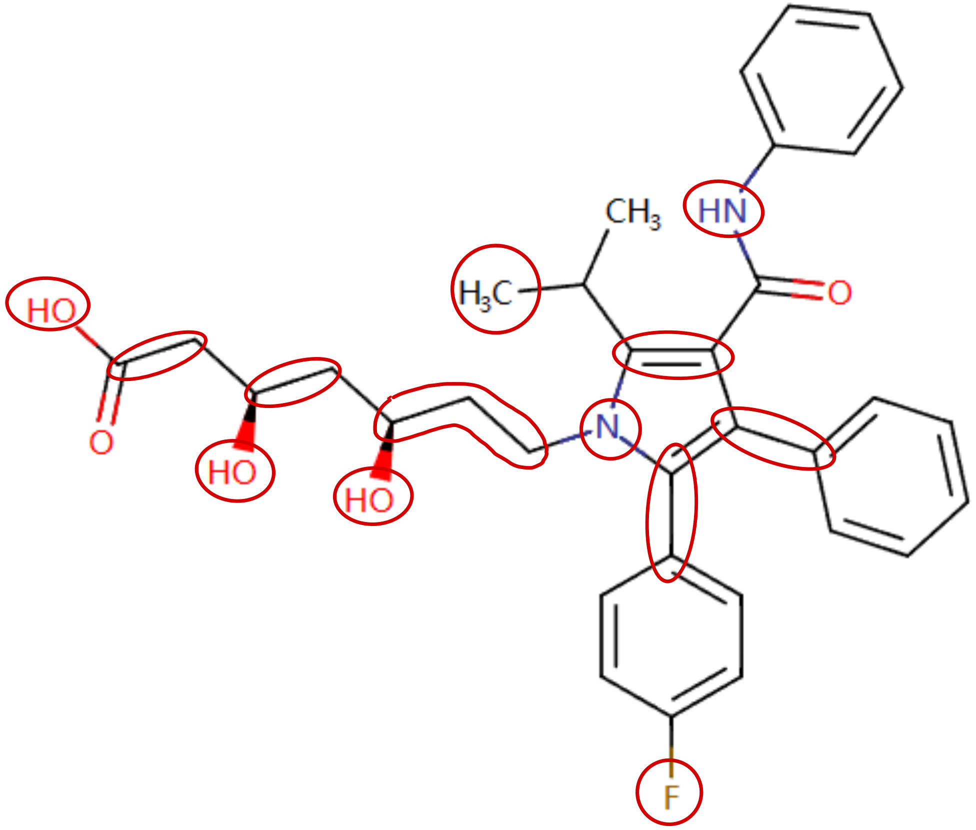

0.04-0.03

rosuvastatin calcium CC(C)c1nc(N(C)S(C)(=O)=O)nc(-c2ccc(F)cc2)c1C=CC(O)CC(O)CC(=O)O

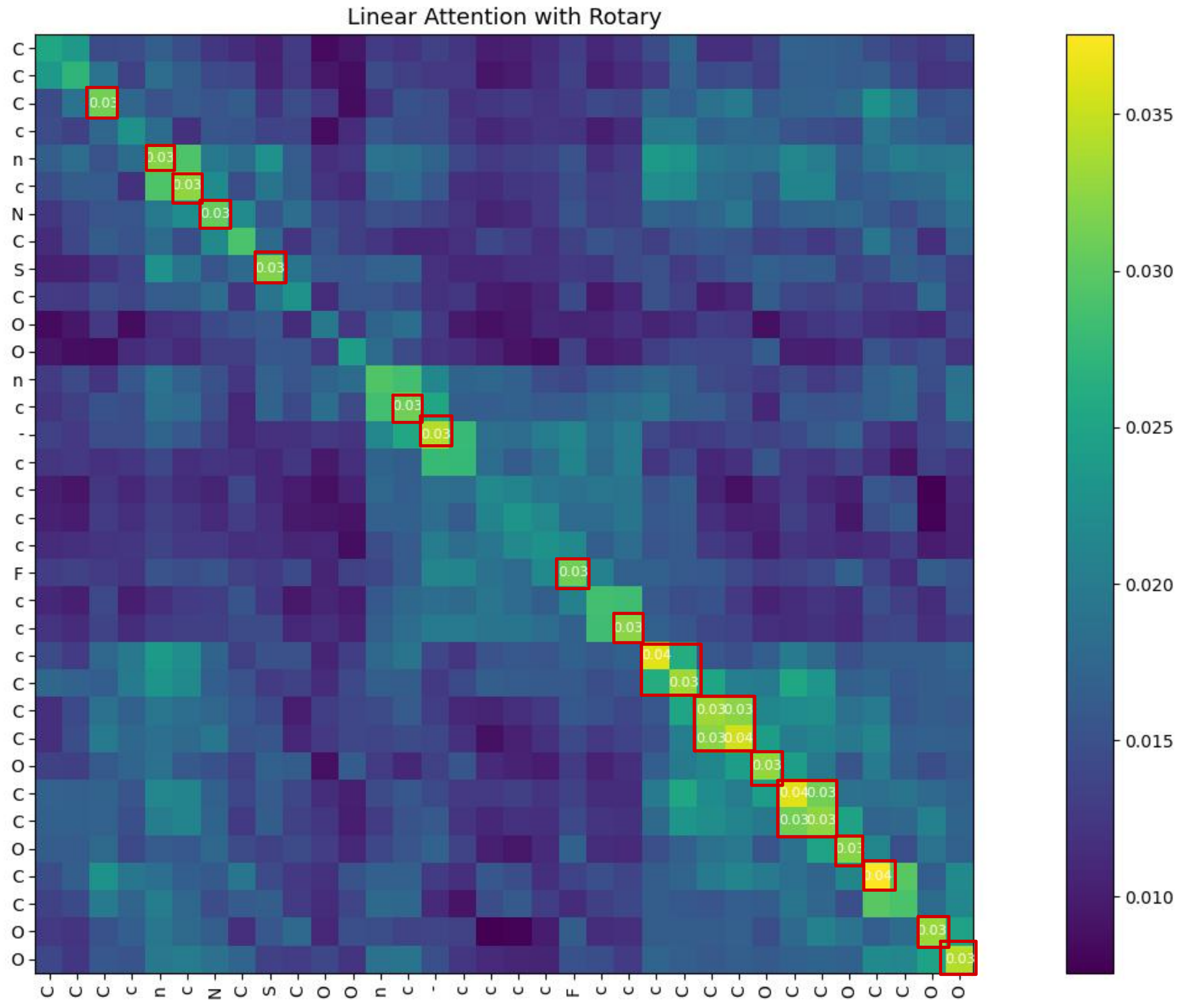

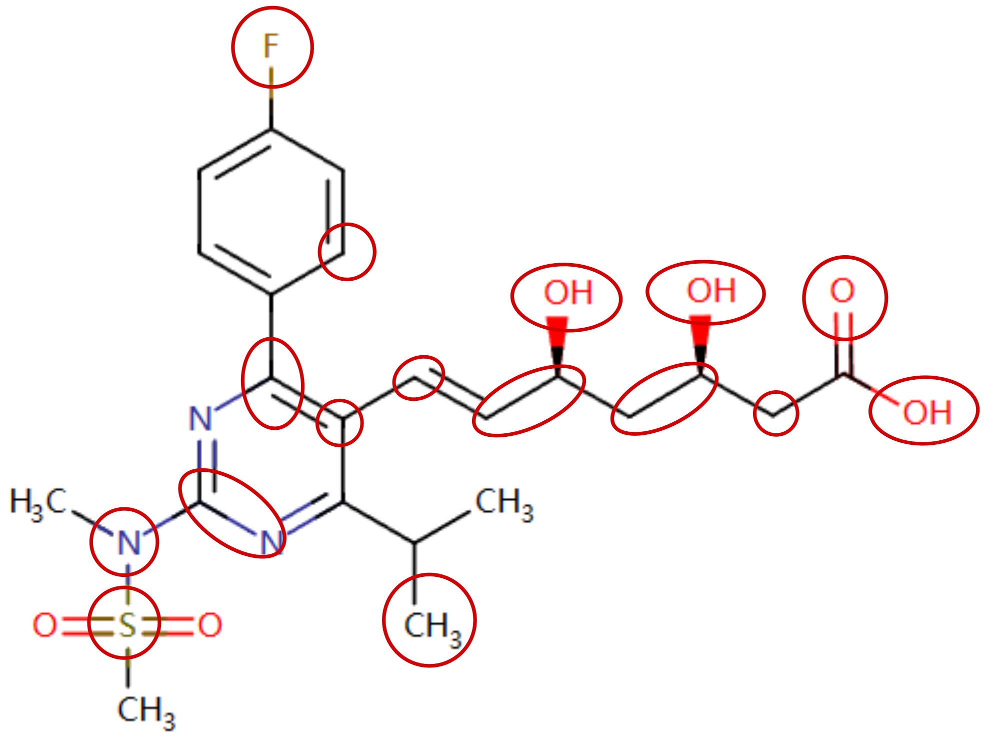

0.04-0.03

pitavastatin calcium O=C(O)CC(O)CC(O)C=Cc1c(C2CC2)nc2ccccc2c1-c1ccc(F)cc1

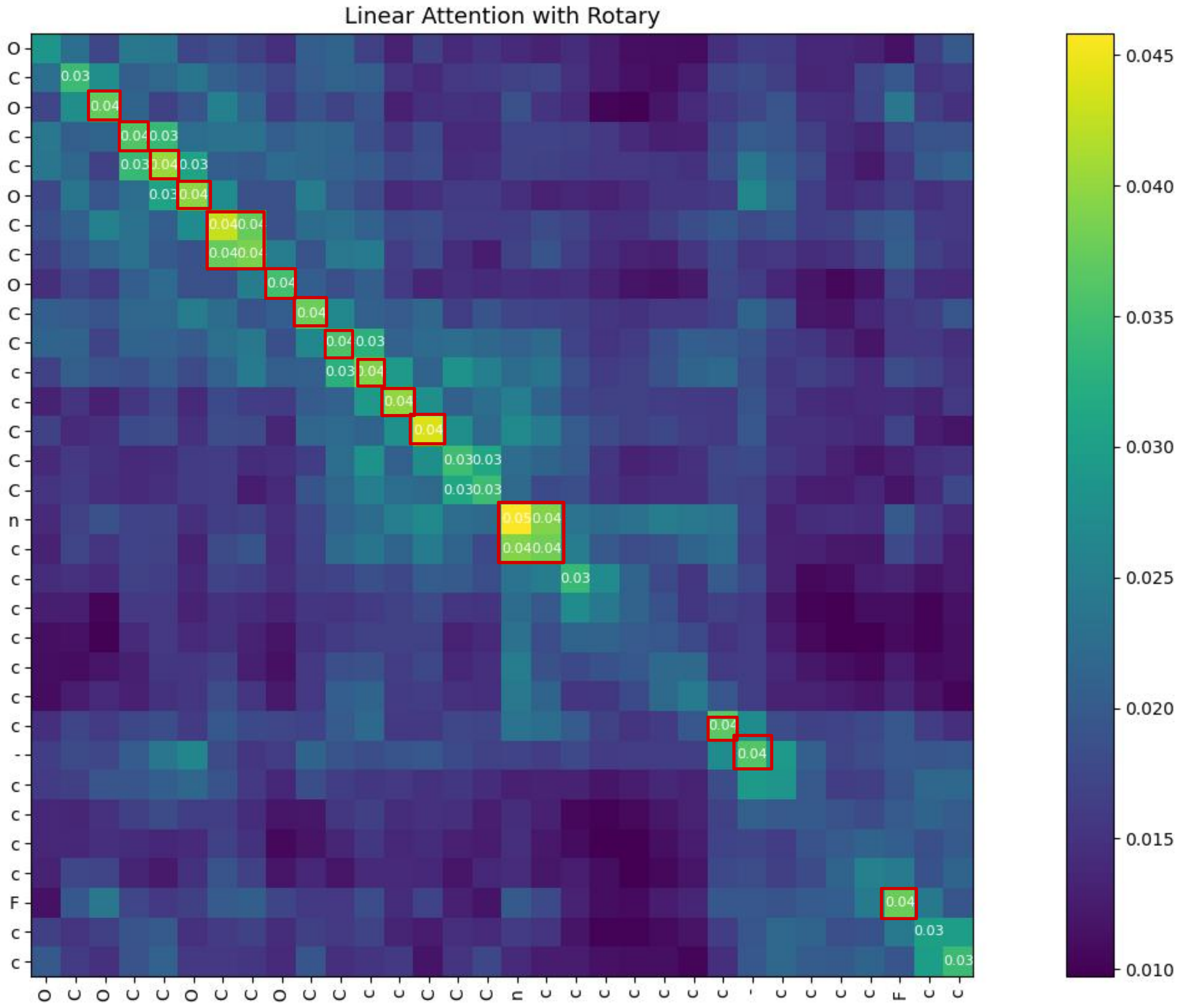

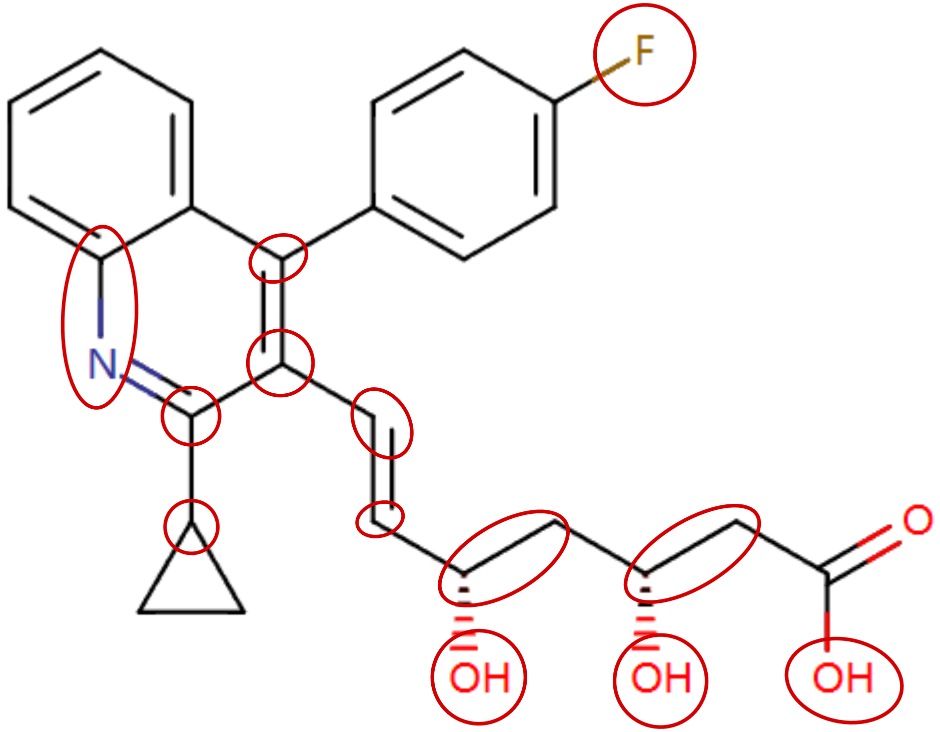

0.05-0.04

simvastatin

CCC(C)(C)C(=O)OC1CC(C)C=C2C=CC(C)C(CCC3CC(O)CC(=O)O3)C21

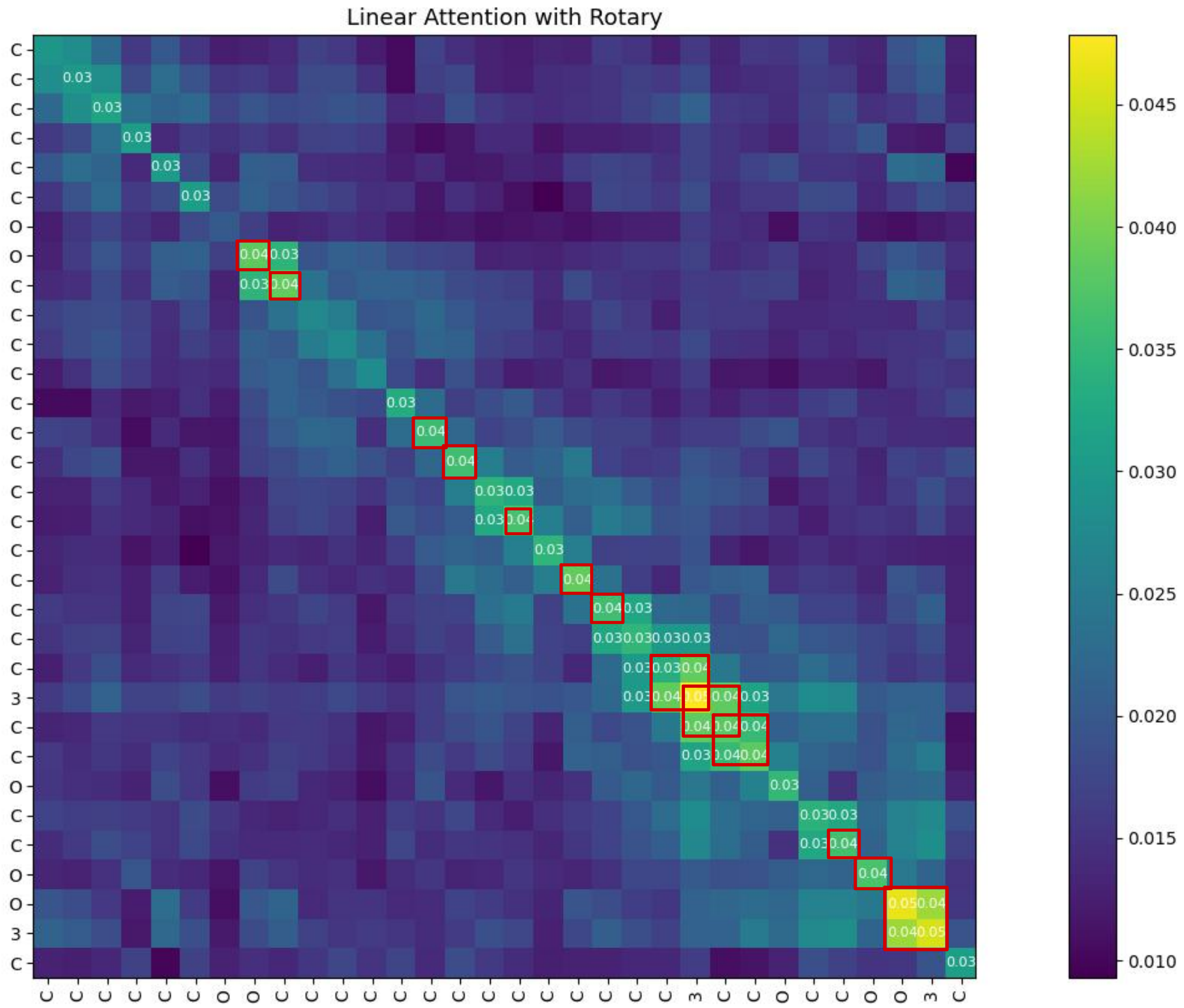

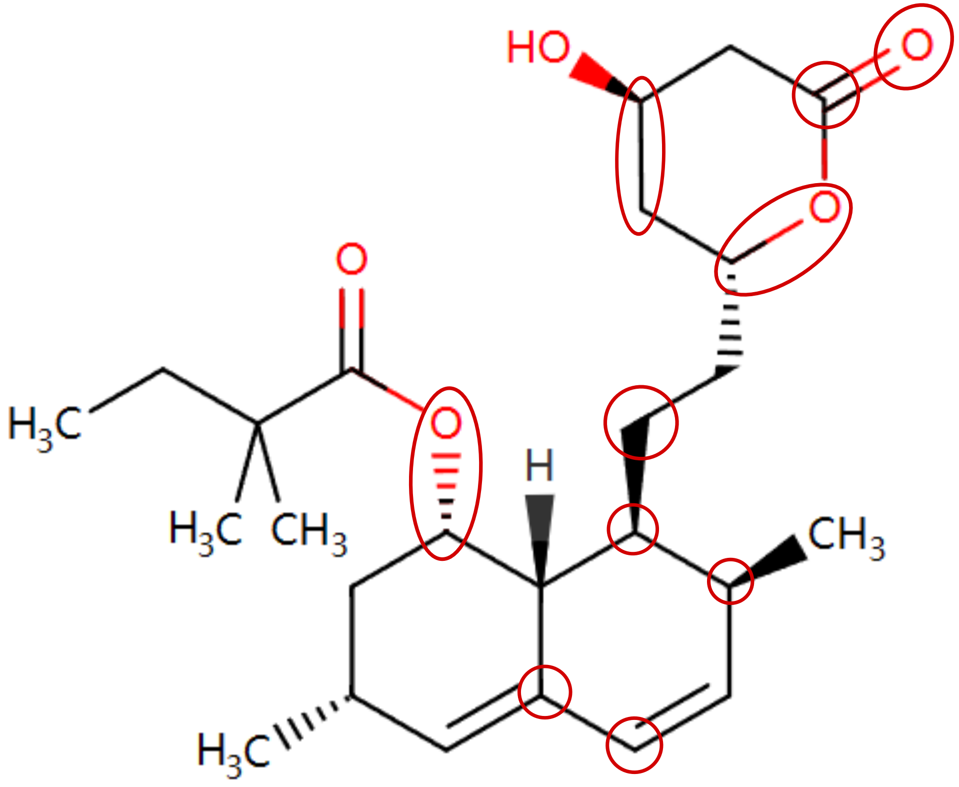

0.05-0.04
