# Supplementary figures and images for: Advancing Adverse Drug Reaction Prediction with Deep Chemical Language Model for Drug Safety Evaluation
Source: Int J Mol Sci. 2024 Apr 20;25(8):4516. doi: 10.3390/ijms25084516 (PMC11050562; doi:10.3390/ijms25084516)

phenytoin

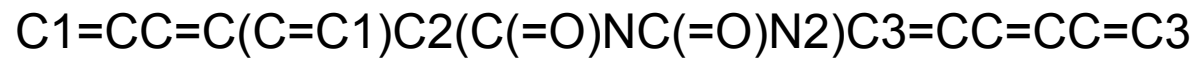

Linear Attention with Rotary

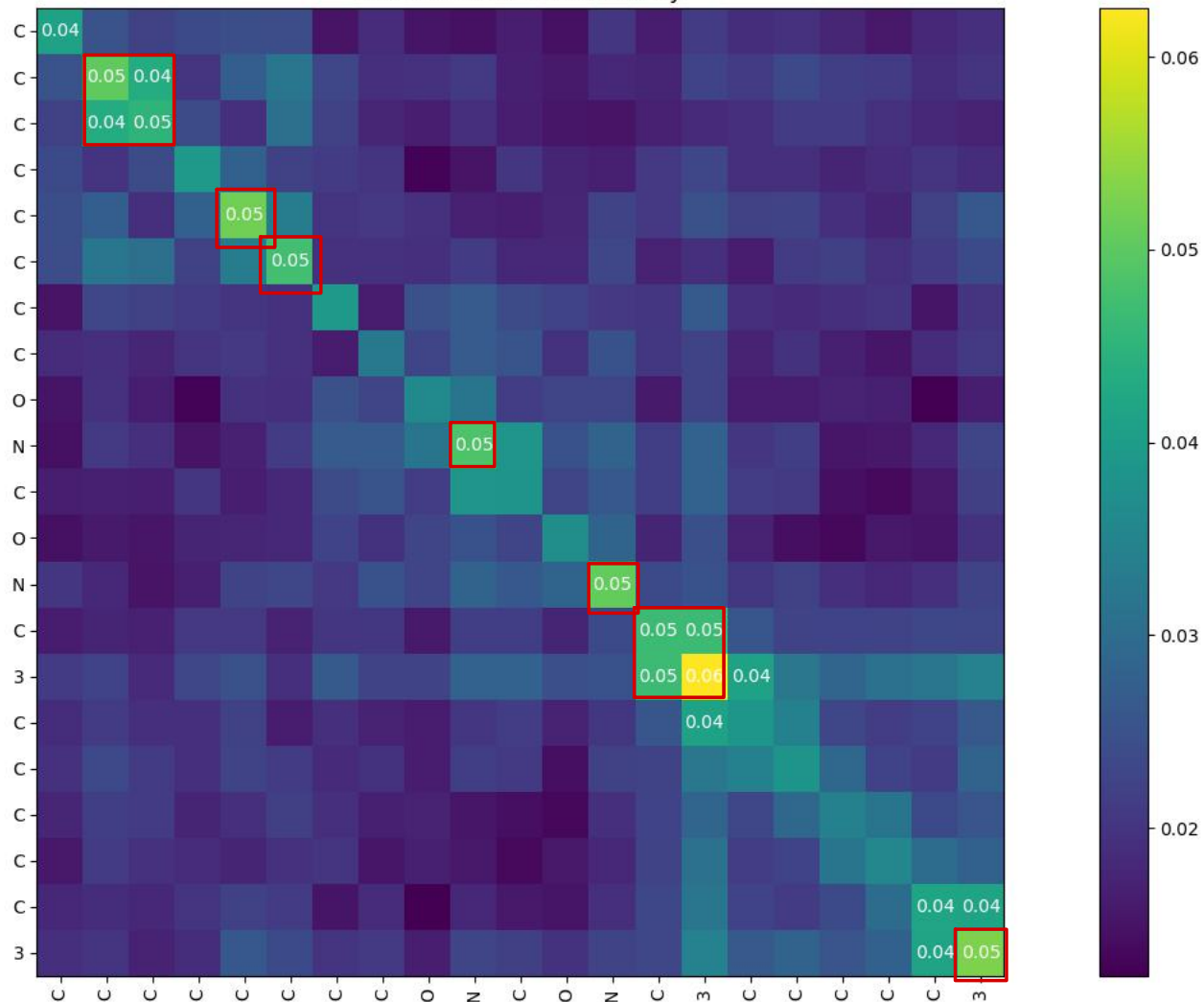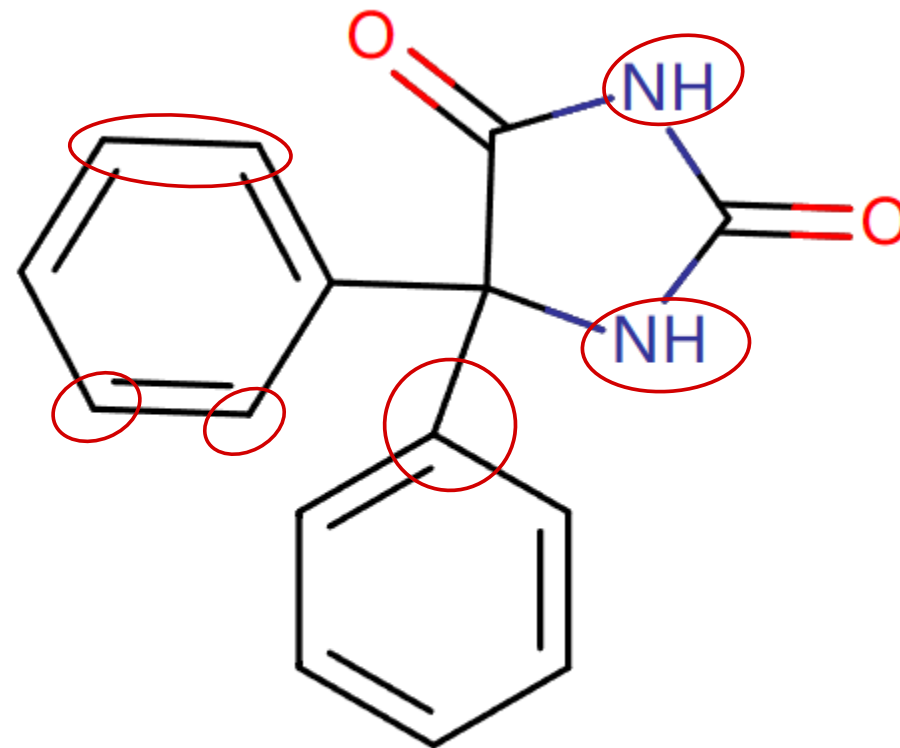

0.06-0.05

valproate

CCCC(CCC)C(=O)O

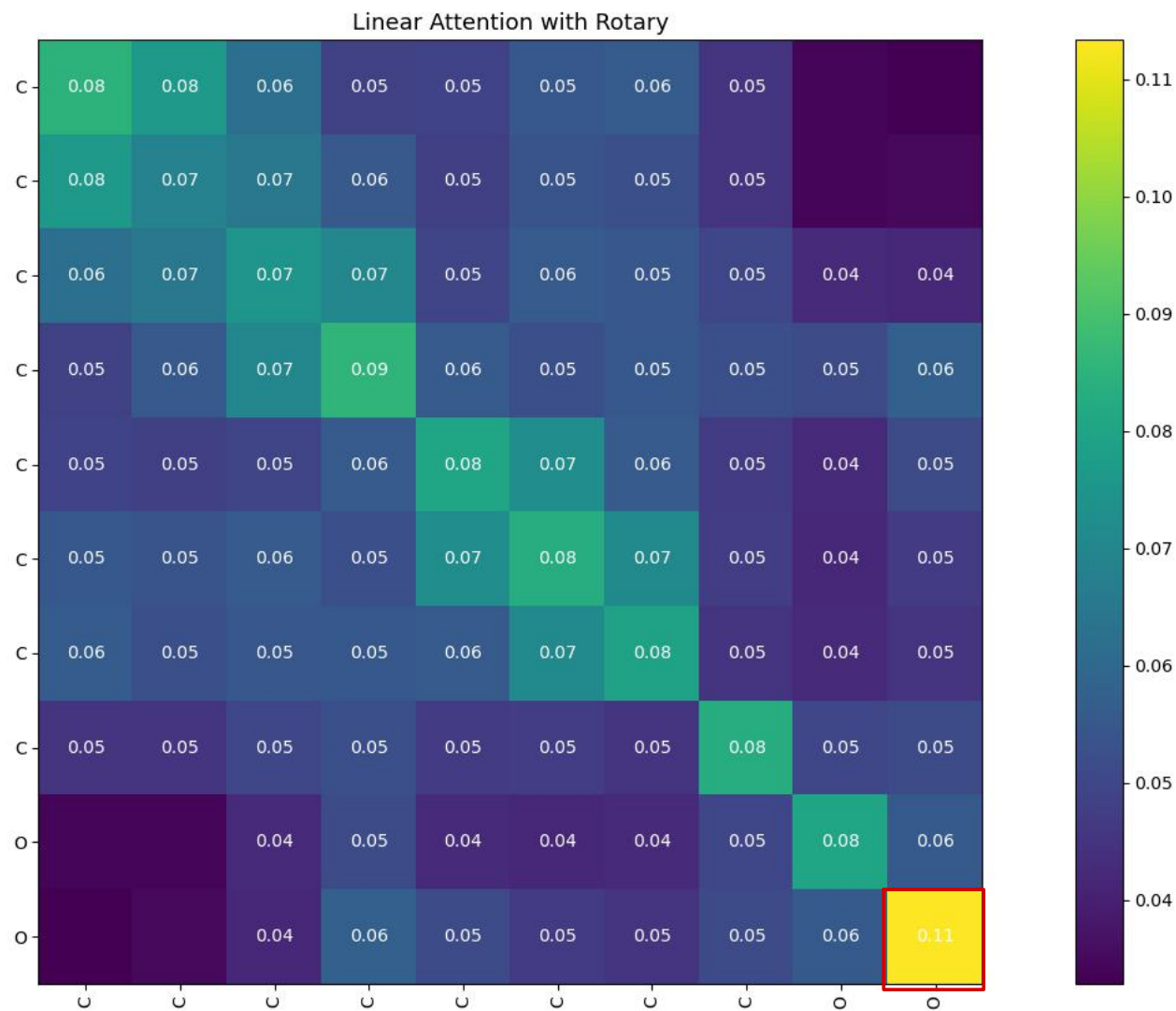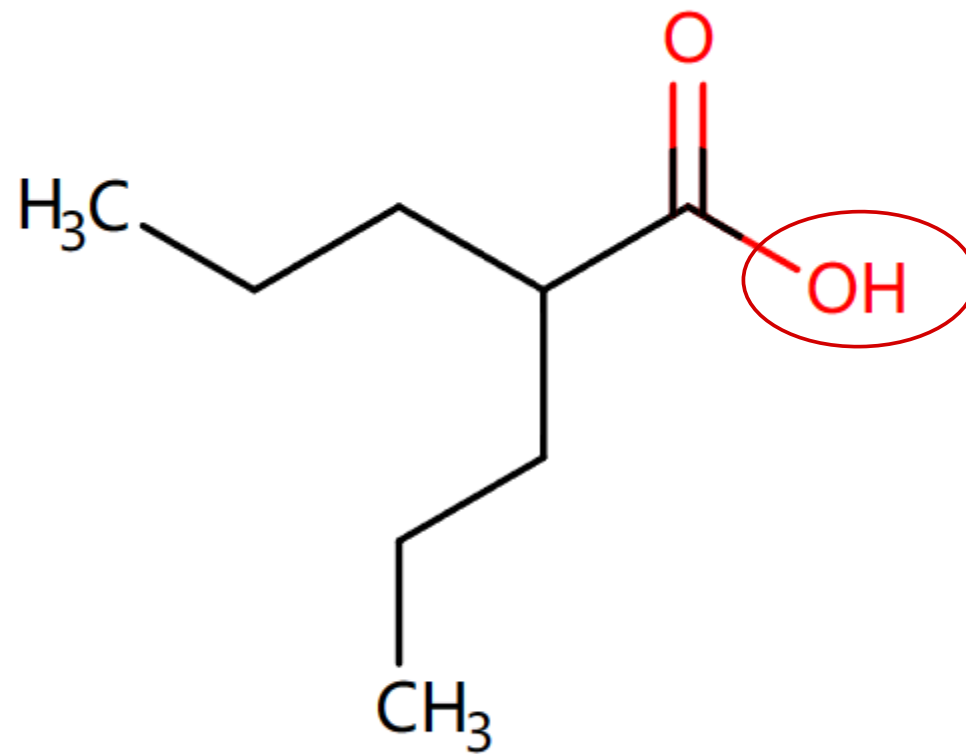

Supplement: Supplementary file 1 [file ijms-25-04516-s001.zip › Supplementary File S2.pdf]
